# The Effect of Bottleium, a Bottle-Type Aquarium, Ownership on Community-Dwelling Older Adults

**DOI:** 10.1093/geroni/igaa057.380

**Published:** 2020-12-16

**Authors:** Mai Takase, Ryogo Ogino, Keishiro Yoshida, Hikari Kusunoki, Tetsuya Kenmochi

**Affiliations:** 1 The University of Tokyo, Tokyo, Tokyo, Japan; 2 GEX International Corporation, Higashiosaka, Osaka, Japan

## Abstract

The pet ownership of ornamental fish acts positively towards the well-being of older adults. A Bottleium is an aquarium that uses a glass bottle instead of a tank. Its small size allows older adults to own ornamental fish as a pet. In this study, the effect of Bottleium ownership on the daily life of community-dwelling older adults was explored. A three-hour workshop to build one’s own Bottleium (size: 11cm×11cm×20cm) was hosted at Toyoshikidai housing complex (Kashiwa, Japan). An ornamental fish and a freshwater snail were added to each bottle. Semi-structured interviews were conducted with participants one month after taking the Bottleium home (N=25). The Bottleium ownership acted as a stimulus to older adults. The effects on an individual were “trigger of conversation” and “development of responsibility.” Participants living alone treated the fish as their companions and had light conversations. They carefully looked after the fish as would a pet owner. Furthermore, the Bottleium facilitated “interpersonal interactions.” Basic information was provided during the workshop, but participants assisted each other in complementing the information, and they even visited each other’s residences to observe the fish. Older adults who do not own the Bottleium also visited for this purpose. Two cases were reported where participants looked after each other’s fish in times of hospitalization. The interpersonal interactions might have resulted from the nature of the housing complex, as participants were already familiar with each other. Similar phenomena could be anticipated at places such as apartments and nursing homes.

